# Postbiotics as Multifunctional Bioactives: Mechanistic Insights and Translational Applications in Host Physiology and Microbial Ecosystem Modulation

**DOI:** 10.3390/microorganisms14061230

**Published:** 2026-05-30

**Authors:** Nidhisha Babysulatha Sasidharan, Sreetha Hely, Subin John, Kalyani Arun, Nandhana Joy Raveendran, Ghanta Rishitha, Sreya S. Kumar, Kongot Abhilash Nair, Sanjay Pal, Damu Sunilkumar, Bipin G. Nair, Vidhya Prakash

**Affiliations:** 1 School of Biotechnology, Amrita Vishwa Vidyapeetham, Kollam 690525, Kerala, India; 2 The Ohio State University Wexner Medical Center and Comprehensive Cancer Center, Columbus, OH 43210, USA; sunilkumar.3@osu.edu

**Keywords:** postbiotics, immunomodulation, cancer, gut–skin axis, wound healing, MAPK, short chain fatty acid (SCFA), neurodegeneration

## Abstract

Postbiotics are increasingly recognized as a predominant group of biotherapeutic agents sourced from the microbial secretome, offering functional benefits, while circumventing the safety concerns associated with the application of live microbial consortia. These microbial derivatives are emerging as promising approaches for tackling complex diseases, encompassing cancer, autoimmune diseases, and metabolic disorders, through modulation of host cell signalling pathways, including G protein-coupled receptors (GPCRs), the NF-κB (Nuclear Factor Kappa B) pathway, and epigenetic regulatory pathways. Besides systemic effects, postbiotics may also have localized effects, such as epithelial regeneration, modulation of fibroblast functions, and control of collagen remodelling. Eventually, the scale-up in the production of postbiotics has initiated new avenues in improving sustainable agriculture and environmental biotechnology. This comprehensive review attempts to integrate mechanistic insights and translational applications, highlighting the therapeutic potential of postbiotics across biomedical and ecological domains. These observations could pave the way to bridge the gap between microbiome regulation, precision medicine, and sustainable biotechnology, thereby positioning postbiotics as a versatile tool addressing some of the most pressing health and sustainability challenges of the 21st century.

## 1. Introduction

The human gut microbiome is a complex mixture of microorganisms which play an essential role in maintaining host health and physiological homeostasis. One of the major developments in this field is the growing awareness on the reciprocal communication between the central nervous system and the gut microbiota termed the ‘gut-brain axis’. This interaction synergistically impacts gut microbiota encompassing metabolic functions, nervous system development, immune system signalling, and production of metabolites [1,2]. Such insights represent a shift from pathogen-centric paradigm, focused on microbial elimination towards a more comprehensive understanding of the role of beneficial microbes in maintaining the balanced state of the host [3]. However, the increasing use of nonselective antimicrobial therapy has led to the disruption of beneficial microbial communities and their complex interrelationships with one another and with their host’s microbial and environmental systems, highlighting the necessity for alternative approaches that preserve the microbial community while targeting the pathogenic organisms [4]. These disadvantages highlight the necessity for alternative approaches that target pathogens but do not impair beneficial microbial functions, thereby developing a sustainable approach for improving human health.

To make certain conceptual clarity, this review adopts the definition proposed by the International Scientific Association for Probiotics and Prebiotics (ISAPP, 2021), which defines postbiotics as “a preparation of inanimate microorganisms and/or their components that confers a health benefit on the host” [5]. The definition highlights postbiotics as non-viable and clearly distinguishes them from live probiotics. Traditionally known as live microbes, research indicates that most of the health benefits associated with probiotics are derived from the secretion of microbial metabolites [6,7,8]. These metabolites, or compounds, have been referred to as postbiotics and include numerous structural and metabolic products such as acetic acid, propionic acid, butyric acid, large and small molecular weight proteins and glycoproteins, short-chain fatty acids (SCFAs), organic acids, bacteriocins, enzymes, exopolysaccharides, extracellular vesicles, and fragments of bacterial cell walls [9,10]. Recent evidence suggests that the gut microbiome does not serve merely as a passive entity colonizing in the intestine or establishing a microbial population within a host but rather plays an essential role in many of the physiological, metabolic, and immunological functions within the human host by actively producing SCFAs. In parallel, the gut microbiota also regulates bile acid metabolism, wherein primary bile acids synthesized from cholesterol in the liver are enzymatically deconjugated and converted into secondary bile acids (such as deoxycholic acid and lithocholic acid) by microbial bile salt hydrolases and 7α-dehydroxylation pathways, thereby influencing host metabolic and immune signalling pathways [11,12,13,14]. When compared with live probiotics, postbiotics offer several advantages, including improved safety, better physico-chemical stability, and greater predictability in therapeutic effects, making them potential agents to eliminate pathogenic microbial translocation and horizontal gene transfer [15]. Recent technological development in microbial metabolomics enabled the identification of novel postbiotic compounds that influence essential molecular mechanisms, including host signalling, immune responses, epithelial integrity, and antimicrobial defence mechanisms. In this context, the focus of the present review is to evaluate the influence of postbiotics on selected scientific domains, emphasizing its involvement in host–microbiota interactions, immune modulation and translating it to practical use in medicine. Supporting this, recent studies highlight the translational potential of postbiotics in treating human disease through increasing numbers of clinical studies, showing their efficacy over multiple disease conditions [16]. For strengthening the translational significance, postbiotics are also identified as potential therapeutic agents for the treatment of metabolic disorders through modulation of the key biochemical pathways that are involved in inflammation, insulin resistance and lipid metabolism [17].

In addition to clinical applications, postbiotics have recently established themselves as well-diversified biological agents for the sustainable management of ecosystems. In agricultural systems, postbiotic compounds are proven to serve as efficient biostimulators and biocontrol agents by promoting plant growth and disrupting quorum sensing of pathogens and biofilm formation [18]. Moreover, they also contribute to environmental remediation through enzymatic breakdown of the biofilms produced within the industrial aquatic environment, minimizing risks of toxicity associated with microbial release in the environment [19]. Collectively, this review aims to provide a broad framework implying advances in the field of postbiotics beyond conventional definitions by presenting them as a discrete molecular entity with a focus on host health and ecosystem management. While most of the existing literature focuses on the detailed studies of the postbiotic compounds themselves, this work aims to explore their applications and efficacies across wound care, agriculture, skin infections, bioremediation, immunotherapy, quorum sensing, and next-generation vaccination, highlighting the importance of postbiotics in clinical and industrial applications.

## 2. Postbiotics as Emerging Wound Therapeutics: Antivirulence Modulation and Promotion of Regenerative Healing

Wound postbiotic therapies find applications such as antimicrobial and anti-quorum-sensing agents to negate infection-induced barriers to wound healing. Cell-free supernatant (CFS), cell lysates, and heat-killed cultures could potentially inhibit both planktonic and biofilm bacteria, as well as disrupt QS and decrease intracellular survival of pathogens without solely depending on bactericidal activity. For instance, *Limosilactobacillus reuteri* EIR/Spx-2 CFS suppressed pathogen growth, disrupted AHL-based QS, and diminished biofilm integrity as measured by QS-based reporter assays and biofilm biomass analysis [20]. Similarly, *Latilactobacillus curvatus* BGMK2-41 lysate significantly lowered intracellular *Staphylococcus aureus* (USA300 and diabetic-foot isolates) in keratinocytes and ex vivo skin by inducing host antimicrobial effectors (DEFB4, ANG, RNASE7, CAMP) and restoring tight-junction proteins (OCLN, TJP1 [21]. Following the same paradigm, a postbiotic agent obtained from *Lactobacillus acidophilus* (strain Scav) exhibited efficacy comparable to or surpassing that of ciprofloxacin in a murine wound infection model with *Pseudomonas aeruginosa*. Bacterial load was reduced in the tissue, thereby preventing the biofilm persistence in the wound environment, thereby establishing the limitations of traditional antibacterial medications in biofilm-ridden environments [22]. Likewise, *P. aeruginosa* anti-biofilm/antivirulence activities of cell-free supernatants of lactobacilli are retained even with minimal bactericidal killing. Thus, the pH irresponsiveness of these compounds in inhibiting virulence factors and biofilm formation of *P. aeruginosa* has been established. In vitro testing has been validated in *Galleria mellonella* infection models [23]. Although QQ mechanisms are thought to exert relatively less selective pressure compared with traditional antibiotics, the possibility of adaptive responses cannot be excluded. It is possible that bacteria might adapt to the selection pressure by developing resistance through changes in signal molecule structure or receptor binding sites, alternative signal pathways activation, or reduced requirement for QS-regulated pathways for pathogenesis, which could attenuate the efficacy of QQ-based interventions [24]. Topical heat-killed *Lactobacillus plantarum* KB131 enhanced wound healing by stimulating C-type lectin receptors and inducing a CARD9 (Caspase Recruitment Domain-containing protein-9)-dependent immune-modulating pathway that includes NF-κB signalling, which promoted early M2 macrophage recruitment, angiogenesis (↑CD31), and myofibroblast activation (↑α-SMA) to enhance wound resolution and tissue regeneration [25]. The resolution phase of wound healing is critically monitored by M2-polarized macrophages by exerting anti-inflammatory effects, promoting tissue remodelling, and facilitating extracellular matrix deposition. Their recruitment helps change the wound microenvironment from a pro-inflammatory to a reparative state, thereby enhance healing and improves tissue regeneration. Heat-killed *L. plantarum* GMNL-6 and *L. paracasei* GMNL-653 promote fibroblast proliferation, accelerate early extracellular matrix (ECM) remodelling via upregulation of matrix metalloproteinase-1 (MMP-1), and later limit fibrotic over-deposition. Mechanistic dissection shows that purified lipoteichoic acid (LTA) from these strains reproduces the anti-fibrogenic phenotype in TGF-β-stimulated fibroblasts, implicating LTA–host receptor interactions (likely TLR2 and/or scavenger/C-type lectin pathways) that modulate TGF-β signalling and downstream collagen homeostasis [26].

Supporting the importance of non-viable microbial structures, extracellular vesicles (EVs) released by *Lacticaseibacillus rhamnosus* were shown to possess distinct proteomic signatures enriched in signalling and stress-response proteins, conferring significantly stronger antioxidant and anti-inflammatory activity. These probiotic strain-derived vesicles further act as nano-signalling messengers between microbial components and host cells, thereby enhancing postbiotic effects. These observations reinforce their role as specialized postbiotic effectors in skin and wound repair [27]. Mechanistically, the “non-live” active moieties in heat-killed preparations and soluble lactic acid bacteria (LAB) lysates fall into three molecular classes that cooperate to produce anti-biofilm and antivirulence outcomes: (A) cell wall glycoconjugates (LTA, peptidoglycan fragments, surface proteins) that act as pattern ligands to reprogram innate immune cells and increase antimicrobial peptide (AMP) expression in keratinocytes, (B) small heat-stable peptides and bacteriocin fragments that inhibit adhesion or destabilize biofilm matrix components without necessarily killing planktonic cells, and (C) low-molecular-weight metabolites (organic acids, short peptides, antioxidant molecules) that interfere with quorum sensing and lower oxidative stress in host cells. Together these effects reduce biofilm biomass, inhibit adhesion and dispersal, and attenuate virulence factor expression rather than relying on broad-spectrum bactericidal killing [28]. Fractionation studies further demonstrate that postbiotic effects are achieved through synergism among different molecular fractions. Specifically, the low-molecular-weight fraction (≤3 kDa) is made up of organic acids (e.g., lactic and acetic acid) and small soluble antimicrobial compounds, while the high-molecular-weight fraction (>3 kDa) consists of bioactive proteins, enzymes, and larger immune-modulating peptides. Reconstituting these fractions regains antibacterial, anti-biofilm, and immunomodulatory activities against *Pseudomonas aeruginosa*. [29]. At the biofilm/matrix level, heat-killed-derived proteases and small peptides can enzymatically or competitively degrade extracellular polymeric substances (EPS), reduce polysaccharide–protein crosslinks, and expose bacteria to host AMPs and phagocytes, while immunomodulation (↑AMPs such as β-defensin, RNase7) in keratinocytes reduces intracellular reservoirs of pathogens. The combined result is a lowered pathogen load and biofilm destabilization that reduces selection pressure for classical antibiotic resistance [30]. Consistent with this model, combined antimicrobial photodynamic therapy followed by *L. acidophilus* CFS resulted in synergistic disruption of MRSA biofilms, severe membrane damage, and suppression of bacterial re-adhesion, demonstrating how postbiotics can be integrated with non-antibiotic modalities to dismantle resilient wound-associated biofilms [31].

Simultaneously, postbiotics directly stimulate host reparative programmes, enhancing fibroblast/keratinocyte proliferation and migration, modulating ECM turnover, promoting angiogenesis, and resolving inflammation by defined signalling pathways. *L. reuteri* CFS enhanced fibroblast proliferation, migration, and collagen-I expression and reduced intracellular ROS, supporting granulation formation and preventing oxidative senescence [32]. For translation, formulation, and delivery count, topical cream, hydrogel, and biopolymer dressings preserve and maintain postbiotic activity and provide mechanical/ECM support. Cold-cream formulations containing postbiotics from *L. fermentum*, *L. reuteri* and *B. subtilis* natto enhanced hydroxyproline (collagen) and improved histology in rat wounds [33]. Hydrogels formulated with recombinant lactonases (Ahl-1) diminished *Pseudomonas aeruginosa* burdens and improved burn healing in mice, illustrating the topical delivery of enzymatic quorum quenching (QQ) for robust antivirulence outcomes [34]. Bacterial extracellular vesicles are another scalable and stable delivery platform; *L. rhamnosus*-derived EVs promoted re-epithelialisation and angiogenesis in full-thickness wound models by miRNA-driven host metabolic reprogramming, extending postbiotic design beyond soluble fractions [35]. Postbiotic formulations based on oral/systemic delivery systems containing the CFS of *Bacillus amyloliquefaciens* fermentation, rich in bioactive lipopeptides (surfactin, iturin, fengycin), antimicrobial peptides, and exopolysaccharides, combined with postbiotics of 127 whey fermentation products (organic acids and bioactive peptides), demonstrate gut–skin axis benefits, restoring tight junctions modulating microbiota, and reducing systemic inflammation, which can indirectly aid cutaneous repair [36,37,38].

Studies elucidating the effectiveness of oral supplements on skin conditions usually rely on phenotypic markers. Oral administration of antioxidants such as reduced and oxidized glutathione, γ-glutamylcysteine, and cysteinylglycine (cystine peptides) indicates suppression of UV-B induced erythema, characterized by studying pigmentation and melanin [39]. Consumption of glucoraphanin and curcumin supplements orally shows a marked increase in the cytoprotective gene NQO1, and a subsequent decrease in pro-inflammatory cytokines (HO-1, IL-1B, TNFA) present in the skin [40]. However, a prominent research gap in the characterization of the gut–skin axis still remains the lack of studies focused on using systemic biomarkers to monitor change in skin health. With established therapeutic properties and antimicrobial effects mediated by these metabolites, postbiotics have proven their worth by demonstrating their ability to combat microbes, decrease their virulence, modulate the immune system, and promote tissue healing. Although challenges related to formulation compatibility, phage stability, and controlled release must be carefully addressed, particularly given the sensitivity of phages to both pH and enzymatic environments, recent advances in hydrogel engineering, compartmentalized delivery, and sequential-release designs offer feasible solutions for complex and infected wounds.

## 3. Postbiotics as Microbial Ecosystem Modulators: Competitive Pathogen Control and Commensal Niche Support

Most postbiotics targeting antimicrobial communication act through either enzymatic quorum-quenching (QQ) or signal antagonism in an attempt at weakening the expression of pathogen virulence factors without inducing complete cell death. This could render a lower selective pressure for resistance and allow survival of commensal organisms. For instance, multiple *Bacillus* isolates exemplify enzyme-mediated QQ. *Bacillus velezensis* D-18 encodes a lactonase (ytnP homologue) that hydrolyses long- and short-chain N-acyl-homoserine lactones (AHLs), which was validated by *Chromobacterium violaceum* biosensor assays. Similarly, AHL degradation suppressed *Vibrio anguillarum* biofilm formation and virulence traits, thereby reducing pathogen fitness and indirectly facilitating niche space for commensals [41]. A related YtnP lactonase (YtnP-ZP1) from *B. paralicheniformis* ZP1 hydrolyzes AHLs produced by *Pseudomonas aeruginosa*, reducing swarming, elastase activity, and biofilm biomass without bactericidal effects where in, in vivo zebrafish trials showed improved survival and synergism with antibiotics for biofilm clearance [42]. Deep-sea *B. velezensis* DH82 similarly produces multiple QQ enzymes (YtnP-type lactonases and likely acylases) that selectively hydrolyse AHLs and downregulate QS regulons in Gram-negative pathogens, linking enzymatic QQ to reduced virulence gene expression and partial biofilm inhibition [43]. Enzymatic quorum quenching (QQ) exemplifies a conserved mechanism: AHL hydrolysis deactivates receptors (LuxR-type or homologues), downregulating virulence and biofilm formation, thereby suppressing pathogens while minimizing off-target effects. Beyond enzymes, postbiotic strategies employ non-enzymatic antivirulence agents and antimicrobial peptides that combine targeted pathogen control with microbiome sparing. Engineered and natural bacteriocins act by receptor-mediated targeting or membrane disruption, producing strong anti-pathogen activity while limiting collateral damage. For instance, the hybrid bacteriocin H1 (N-terminal of Enterocin K1 fused to C-terminal of Enterocin EJ97) recognizes the membrane protease receptor RseP in *Staphylococcus haemolyticus*, causing membrane destabilization and potent killing of both planktonic and biofilm cells. When used in a three-component cocktail with micrococcin P1 and garvicin KS, H1 eradicated biofilm-embedded cells and prevented resistance emergence, illustrating how receptor-specific bacteriocins can suppress pathogens while preserving broader community structure [44]. To further compliment these molecular tools, engineered live biotherapeutics (eLBP’s) that secrete bacteriocins (e.g., modular *E. coli* EntA/EntB secretion systems) and synthetic ecological circuits facilitate amensalism through toxin secretion conditioned on competitor-derived cues offering a programmable framework to maintain target suppression and stable coexistence [45,46]. Simultaneously, QS-regulated bacteriocin circuits in LAB and *Streptococcus thermophilus* (BlpC system) showed how endogenous quorum signals tune bacteriocin output to population context, balancing competitive exclusion and self-limitation, a mechanistic basis for commensal support via density-dependent antimicrobial release [47]. Many postbiotics combine dual modalities which are QQ enzymes plus secreted antimicrobial or immunomodulatory metabolites producing additive or synergistic effects on pathogen suppression and commensal support. *B. velezensis* DH82 is a classic example, where apart from robust YtnP-type lactonase activity (broad substrate profile, thermostable kinetics), DH82 secretes extracellular peptide-based postbiotic compounds which displayed antibacterial action against *Vibrio parahaemolyticus*. In *Litopenaeus vannamei* shrimp challenges, DH82 application reduced *Vibrio* colonization, restored host immune enzyme activities (ACP, AKP, SOD), and lowered tissue damage without changing lysozyme, indicating targeted pathogen suppression together with host/commensal resilience [48]. Similarly, topical application of recombinant lactonase formulations (Ahl-1 hydrogel) to burn infections caused by multidrug-resistant *Pseudomonas aeruginosa* significantly reduced bacterial loads in tissue, impeded systemic dissemination, enhanced survival, and accelerated reepithelialisation. These studies represent an applied demonstration that enzymatic quorum quenching can translate into clinical and pathological benefits apart from broad-spectrum antimicrobial killing [34]. Experimental evidence reveals that QQ/AHL-hydrolysis approaches selectively attenuate virulence programmes, such as the Las/Rhl/Pqs/Iqs networks in *Pseudomonas aeruginosa*, decrease toxin and exoproduct secretion, weaken biofilm architecture, and collectively reduce pathogen competitiveness, thus favouring recolonisation or persistence of commensals [49].

The LAB-derived postbiotics of *L. plantarum*, *L. acidophilus*, *Lacticaseibacillus casei*, *L. rhamnosus GG* and *Bifidobacterium animalis* subsp. *lactis* exerted wide-spectrum antimicrobial effects against Gram-negative and Gram-positive pathogens, including *Escherichia coli*, *Salmonella typhimurium* and *Staphylococcus aureus*. Mechanistically, pathogen suppression was mediated through a multicomponent postbiotic matrix comprising bacteriocin-like inhibitory substance (BLIS), organic acids, hydrogen peroxide, fatty acids, and oleic acid, which, through their cumulative mode of action, destabilized pathogen membranes and disrupted metabolic processes with minimal selective pressure for resistance. The maximum biosynthesis production dynamics ranged from 24 to 36 h, which reflects a density-dependent and metabolism-dependent biosynthesis mechanism, where lyophilisation enhanced the stability and concentration of the postbiotic compounds [50]. Moreover, postbiotics may work as resistance-modulating agents. *Lactiplantibacillus plantarum* (RP155, RP403, RP225) and *Ligilactobacillus salivarius* RP317-derived postbiotics, when combined with sub-inhibitory β-lactam antibiotics, completely eradicated MDR *Klebsiella pneumoniae* strains by downregulating resistance genes (blaNDM, blaCTX, blaTEM, blaSHV), confirming that postbiotics can sensitize pathogens and reduce ecological selective pressure while sparing commensals [51]. Beyond their antimicrobial and resistance-modulating properties, postbiotics can preserve highly specific immunomodulatory functions following bacterial inactivation. Recombinant *Lactococcus lactis* expressing an IL-6-targeting antibody (ZIL6) remained functional after exposure to ethanol, UV, or gamma irradiation, inhibited up to 78% of IL-6-induced STAT3 (Signal Transducer and Activator of Transcription 3) signalling, and conferred ecological benefits by supporting mucosal integrity and commensal survival [52].

**Figure 1:**
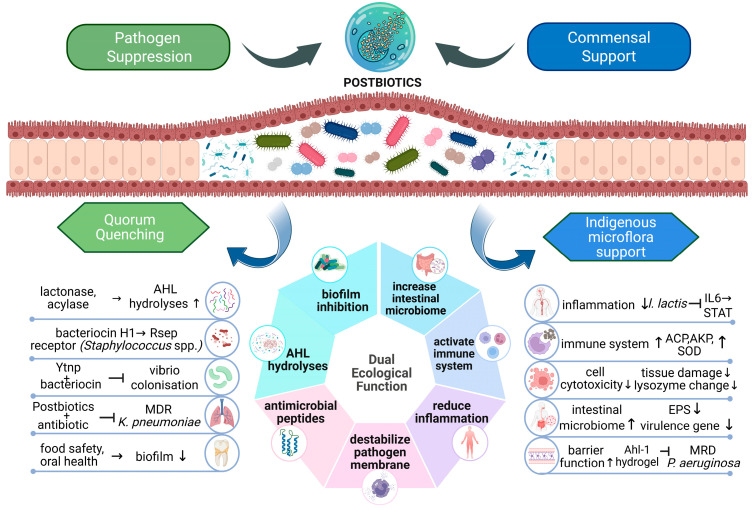
**Postbiotic-mediated pathogen control and commensal niche support:** Postbiotics function in dual roles and inhibit pathogens through quorum quenching (AHL hydrolysis by lactonases/acylases), produce bacteriocins, and promote biofilm disruption thereby limiting colonization of pathogens such as *Vibrio* spp. and multidrug-resistant *Klebsiella pneumoniae*. Conversely, postbiotics enhance beneficial microbiota composition, promote epithelial barrier integrity, and modulate host immunity by regulating IL-6/STAT, thereby increasing defence enzymes (ACP, AKP, SOD), and simultaneously reducing cytotoxicity and virulence gene expression.

In the context of food and cosmetic ecosystems, the postbiotics of *L. rhamnosus* and *L. reuteri* decrease the CFU of *Escherichia coli* and *Staphylococcus aureus* on meat surfaces synergistically. This effect depends on organic acids, phenolic compounds, and antioxidant metabolites and allows for ecologically compatible suppression of pathogens by non-viable microbes [53]. Postbiotics from *Lactiplantibacillus pentosus* B1 inhibited and eradicated biofilms of skin pathogens, including *Staphylococcus aureus*, *Escherichia coli*, *Streptococcus pyogenes*, and *Cutibacterium acnes* without inducing cytotoxicity and hence might offer applications in cosmetics and dermatology [54]. Clinical postbiotics, such as Probio-Eco, restore balance to the intestinal ecosystem by modulating metabolites like succinate, SCFAs, tryptophan derivatives, and cortisol. Such modulation is related to improved symptoms of constipation and positive effects on commensal microbiota by enhanced mucus secretion, barrier integrity, and anti-inflammatory signalling [55]. Moreover, postbiotics formed by pasteurization of dairy LAB inhibit *Staphylococcus aureus* and *Listeria monocytogenes* in cheese models. The preparation methods of postbiotics affect their antimicrobial activity and ecological compatibility [56]. In oral health, it has been observed that postbiotics prevent dental caries by targeting biofilm formation, EPS synthesis, and QS-regulated acid production, suppressing the growth of cariogenic pathogens while maintaining the homeostasis of commensals and host tissue integrity [57]. Collectively, these studies demonstrate that postbiotics are dual ecological modulators that facilitate the suppression of pathogens through antivirulence, anti-biofilm, and targeted antimicrobial activities and foster the survival of commensals while enhancing host resilience and promoting ecological stability in the GI tract, food systems, skin, water, and oral cavity. A schematic representation denoting mediation of pathogen control and commensal support by postbiotics is shown in Figure 1.

## 4. Postbiotics as Immunotherapy Adjuncts: Modulating Drug Responses and Antitumor Immune Activation

Postbiotics provide synergistic effects with medications by enhancing the efficacy of conventional drug therapies. The gut microbiota and their metabolites enzymatically transform drugs, thereby significantly altering drug bioavailability, therapeutic efficacy, and toxicity profiles. This further enhances absorption and metabolism of the drug by modulating cytochrome P450 enzymes, reinforcing intestinal epithelial barrier function, and reducing oxidative stress with such effects widely explored in intestinal epithelial cell systems. These bioactive compounds could therefore reduce the required doses of antibiotics and mitigate antimicrobial resistance pressure [58]. In oncological applications, postbiotics can enhance the efficacy of common chemotherapeutic agents as well as attenuate the adverse effects caused by those agents by means of their anti-proliferative and anti-inflammatory properties, reflecting evidence that currently arises largely from preclinical settings. They regulate inflammatory signalling pathways like NF-κB, MAPK MAPK (Mitogen-Activated Protein Kinase), and NLRP3 (NLR family pyrin domain-containing 3, suppress pro-inflammatory cytokines such as IL-6 and TNF-α (Tumor Necrosis Factor-alpha), and activate pattern-recognition receptors (PRRs), thereby influencing host drug pharmacodynamics [59,60]. A paradigm of improved drug bioavailability on administration of microbial metabolites was observed with a 269.9% increase for omeprazole and a 4.3-fold enhancement for lurasidone as reported in controlled pharmacokinetic investigations [61]. SCFAs reduce the intestinal pH, which supports improved drug solubility and absorption. Beyond pharmacokinetic modulation, postbiotics can mitigate treatment-associated gastrointestinal dysbiosis induced by conventional drug therapy [62,63]. Some studies have shown that SCFAs could reduce drug absorption by activating GPCRs to trigger signal cascades involving PKC (Protein Kinase C) and MAPK pathways that activate transcription factors AP-1 (Activator Protein 1 and Nrf2 (nuclear factor erythroid 2–related factor 2). This consequently upregulates P-glycoprotein, MRP2, and OCT 3 expression, which enhances intestinal drug efflux, resulting in reduced drug absorption [64]. Postbiotics derived from *L. casei*, *L. bulgaricus*, *Enterococcus faecium*, and *Streptococcus thermophilus* showed notable antimicrobial activity against resistant nosocomial pathogens, an effect established through antimicrobial susceptibility and biofilm-based experimental assays. Postbiotics in combination with antibiotics like amikacin and linezolid showed significantly enhanced activity against bacteria, possibly through alteration of bacterial membrane permeability, biofilm disruption, and interference with bacterial communication pathways. The synergistic interactions were observed with respect to *S. aureus*, *E. coli*, *P. aeruginosa*, and *P. mirabilis* [65]. Metformin, used in the management of type 2 diabetes, promoted short-chain fatty acid (SCFA)-producing bacteria (e.g., *Akkermansia muciniphila*, *Faecalibacterium*, *Butyrivibrio* and *Bifidobacterium*) in the gut, an effect consistently observed across both experimental animal models and human clinical cohorts. These bacteria produce SCFAs such as butyrate, propionate, and acetate which are important for maintaining glucose homeostasis in humans. These metabolites activate AMP-activated protein kinase in hepatocytes and peripheral tissues, enhancing insulin sensitivity and reducing hepatic gluconeogenesis. They also stimulate the secretion of glucagon-like peptide-1 (GLP-1) from intestinal L cells, which improves glucose-dependent insulin release [66].

Recent research has also indicated that the gut microbiome, as revealed through both experimental models and patient-based observations, is a vital component modulating effective cancer immunotherapy by its ability to influence overall immune responses via microbial metabolites. For instance, butyrate has been shown to be an effective inhibitor of histone deacetylases (HDACs), triggering epigenetic changes, thereby enhancing CD8^+^ T-cell functionality and IL-12 signalling. Consequently, this contributes to an enhancement of systemic anticancer immunological surveillance and clinical response to the PD-1 and PD-L1 blocking agents with supportive associations emerging from immunotherapy-treated patient cohorts. Therefore, it is posited that there may be a synergistic relationship between butyrate-producing microorganisms and systemic immunity, as evidenced by the consistent correlation between butyrate-producing organisms and better clinical outcomes. This underscores the potential of microbiome-targeted interventions as a precision medicine approach to augment immunotherapy [67]. Cancer therapies alter microbial composition, while the microbiome, in turn, influences drug pharmacokinetics and pharmacodynamics, including the metabolism of agents such as irinotecan and 5-fluorouracil. Specific microbes, notably *Akkermansia muciniphila*, are associated with improved responses to anti-PD-1/PD-L1 immunotherapy, a relationship observed in clinical cohorts of patients undergoing immune checkpoint blockade. Integrating multi-omics and AI-driven microbiome analysis may enable precision oncology strategies, incorporating microbiome modulation approaches such as faecal microbiota transplantation and postbiotics to optimize treatment outcomes [68].

Furthermore, the tryptophan catabolite indole-3-aldehyde (I3A) has been characterized as a pivotal molecular mediator governing the crosstalk between neoplastic cells and the immune system. I3A stimulates the activation of the aryl hydrocarbon receptor (AHR), causing its degradation and upregulation of MHC class I expression in tumour cells. Moreover, I3A-mediated signalling promotes the degradation of the oncogenic driver c-MYC, simultaneously attenuating proliferative capacity and priming the systemic immune response. I3A, an exogenous compound, is consistently synergistic when used together with adoptive T-cell transfer and significantly enhances the overall efficacy of these treatments in melanoma and lymphoma mouse models under preclinical conditions. I3A is classified as a postbiotic, is bioactive and has a capacity to manipulate the TME, and supports the rationale that I3A could have an adjuvant role (in combination) in the use of new Immunotherapeutics [69]. The microbiota is also known to produce Indole-3-Lactic Acid (ILA), a metabolite that plays a crucial role in the regulation of metabolic pathways that are disrupted during colorectal cancer (CRC). In CRC, lower levels of ILA have been associated with the initiation and progression of CRC, while higher levels of ILA demonstrated reverse effects in the AOM/DSS model of CRC. Mechanistically, ILA inhibits the phosphorylation of STAT3, thereby decreasing p-STAT3 activity and subsequently downregulating hexokinase-2 (HK2). Evidence from both cellular experiments and animal models indicates that this process disrupts the Warburg effect and promotes metabolic reprogramming in CRC cells. Notably, these effects occur independently of the aryl hydrocarbon receptor (AhR) signalling axis, highlighting a direct mechanistic link between microbiota-derived metabolites and host cancer metabolism. Collectively, these findings suggest that ILA represents a promising therapeutic candidate for targeting metabolic dysregulation associated with CRC [70].

Metabolomic research now suggests that I3A and ILA serve as promising non-invasive biomarkers for CRC in the context of multi-metabolite assays. For instance, Zhou et al. observed that the concentration of ILA is significantly decreased in feces from CRC patients depending on disease severity, indicating a measurable, disease-linked metabolic shift detectable in a non-invasive biofluid [70]. At the population level, it was observed that although tryptophan alone displays modest biomarker potential, its combination with other metabolites like indoles provides significant diagnostic power [71]. Similar observations were established in plasma-based assays, where tryptophan metabolites have shown to provide excellent diagnostic capacity (AUC ~0.95) in CRC detection [72]. Postbiotic metabolites derived from *L. rhamnosus* and *Bifidobacterium breve*, particularly cell-free supernatants enriched with organic acids (such as lactic and acetic acid), short-chain fatty acids, and bacteriocin-like compounds, exhibit significant anti-proliferative effects on human colorectal cancer HT-29 cells. Cell-free supernatants from these probiotics induce intrinsic, mitochondria-mediated apoptosis by upregulating pro-apoptotic proteins Bax and caspase-3 while downregulating the anti-apoptotic protein Bcl-2. The resulting increase in the Bax/Bcl-2 ratio disrupts mitochondrial membrane integrity, ultimately triggering apoptotic cell death. These findings provide molecular evidence supporting the potential role of postbiotics as adjunct therapeutic agents in colorectal cancer management [73]. Moreover, postbiotic metabolites produced by beneficial *Bifidobacterium* strains have demonstrated potential as adjuvants to enhance chemotherapy efficacy in gastrointestinal cancers. Specifically, N-acetylcysteine (NAC) and tetrahydro-β-carboline carboxylic acid (THC) were shown to improve the therapeutic effectiveness of gemcitabine in pancreatic cancer cells and 5-fluorouracil in colorectal cancer cells with initial insights arising from drug-resistant cancer cell models and extending into preclinical systems. THC exhibits intrinsic anti-proliferative and pro-apoptotic properties, whereas NAC promotes apoptosis in drug-resistant cancer cells. Collectively, these findings highlight the potential of specific postbiotic metabolites as safe adjuvant agents for improving chemotherapy response and overcoming drug resistance [74]. Furthermore, chemotherapy-induced mucositis involving microbiota dysbiosis, epithelial barrier disruption, and increased inflammation have been controlled by microbiota-targeted therapies, including postbiotics, preclinical investigations, alongside limited clinical observations, indicating that these effects involve upregulation of tight junction proteins such as ZO-1 and occludin, reducing microbial translocation and pro-inflammatory cytokines (TNF-α, IL-6). These interventions can alleviate mucositis and improve tolerance to high-dose breast cancer chemotherapy, supporting the potential of personalized microbiota-directed supportive therapies in cancer care [75].

Another advanced system includes the bacterial outer membrane vesicles (OMVs), which could serve as a potential new class of nano systems for cancer immunotherapy. In tumour-bearing animal models, the OMVs express tumour-associated antigens and PAMPs (pathogen-associated molecular patterns) to induce a TLR-mediated immune response directed towards tumour cells, as well as to induce the polarization of tumour-associated macrophages from a M2 immunosuppressive phenotype to a M1 pro-inflammatory phenotype. Thus, OMVs could provide a safer method of delivering drugs in cancer therapy than live bacteria [76]. However, endotoxin-associated toxicity remains a major limitation of bacterial immunotherapy. To address this, genetically engineered outer membrane vesicles (OMVs) derived from lipid A-modified *Escherichia coli* have been developed to generate endotoxin-attenuated immunostimulatory vesicles with minimal systemic inflammatory responses. These modified OMVs can be functionalised with tumour antigens to enhance targeting specificity. Despite the use of such approaches for the controlled activation of TLR4 and stimulation of DCs for proliferation of CD8 T cells, there is an obvious limitation to such approaches on account of safety concerns, which includes presence of endotoxins and variability of immune response, and this can only be overcome through well-designed preclinical and subsequent clinical studies [77].

## 5. Postbiotics in Next-Generation Immunization Strategies

Postbiotics play a major role in supporting conventional therapies as they modulate inflammation, improve, and maintain gut health by promoting the growth of beneficial bacteria. Probiotic-derived EPS and CFS stimulate macrophage activation and cytokine signalling pathways, while SCFAs modulate immune signalling via GPCRs. Postbiotics, therefore, are safer immunomodulatory alternatives while reducing the risk of AMR [78]. They interact with the host immune system through microbe-associated molecular patterns (MAMPs), which engage pattern recognition receptors (PRRs) on immune and epithelial cells, leading to the activation of signalling pathways like NF-κB and MAPK/AP-1. Postbiotics enhance mucosal immunity by increasing IgA production, regulating cytokine profiles, and restoring Th1/Th2 immune balance. They can also compete for the pathogen-binding receptors on enterocytes and result in antibacterial effects [16,79].

Autoimmune conditions like inflammatory bowel diseases (IBD) can stimulate excessive immune response with the release of cytokines and chemokines, as well as damage the intestinal barrier function. As a result, the inflammatory mediators produced would cause tissue damage and increase inflammation. Exopolysaccharides (EPS) of commensal bacteria such as *Lactobacillus*, *Lactococcus*, *Bifidobacterium*, and *Streptococcus* have been shown to attenuate the production of pro-inflammatory cytokines. They also regulate the production of anti-inflammatory cytokines and influence the differentiation of immune cells [80]. SCFAs enhance mitochondrial respiration by lowering oxidative stress to improve mitochondrial function and ATP production. Additionally, structural postbiotics like lipoteichoic acid engage pattern recognition receptors to fine-tune innate immune activation without inducing excessive inflammation [81]. β-Glucans, naturally occurring polysaccharides found in cell walls of microorganisms, have been studied as vaccine adjuvants. They activate immune responses by interacting with pattern recognition receptors (PRRs) on immune cells to trigger signalling pathways NF-κB and MAPK. They also activate the complement system to enhance phagocytosis, thereby improving pathogen clearance [82]. Flagellin could be a potential immunization strategy against obesity and gut inflammation. Elevated faecal flagellin and insufficient flagellin-specific IgA led to motile bacterial encroachment, which could trigger gut inflammation and obesity. Experimental immunization with flagellin induced the production of mucosal flagellin-specific IgA. This limited microbial encroachment, reduced inflammation, and diet-induced obesity [83]. In addition, flagellin injections in mouse models activated TLR5 signalling pathways and type 1 interferon responses to attenuate hepatic fibrosis [84]. These findings highlight how postbiotics can be harnessed as immune-training agents to generate protective host responses and tissue-specific benefits. Additionally, in a study on stress-induced mice, CFSs from *L. plantarum* and *Bacillus velezensis* enhanced immune response by activating natural killer cells. Macrophage activation was stimulated while modulating cytokine expression, which promoted B-cell and innate immune activity [85].

Additionally, postbiotics have been demonstrated to offer benefits in paediatric nutrition. Fermented infant formulas from LAB provide essential compounds for modulating gut health as well as immunity. Clinical trials with fermented formulas from *Lactofidus*, *L. paracasei* CBA L74, and *B. animalis* subsp. *Lactis* BPL1™ HT proved that intestinal homeostasis was improved while the infection rates were reduced significantly. Enhanced mucin production and strengthening of intestinal barriers were also enhanced, substantiating immunomodulatory and anti-inflammatory effects [86]. Fermented milk containing heat-inactivated *L. paracasei* CBA L74 demonstrated protective effects against common infections in children aged 12–48 months (about 4 years). A fermented formula containing *Bifidobacterium breve* C50 and *Streptococcus thermophilus* ST065 demonstrated anti-inflammatory properties on intestinal cells in vitro and reduced the severity of acute gastroenteritis. The mechanisms underlying the efficacy of these formulations appear to involve immune programming through metabolite signalling, which leads to activation of both innate and acquired immune systems [87].

Since postbiotics regulate immune responses as well as maintain mucosal barriers, they exhibit strong immunomodulatory effects against autoimmune diseases like rheumatoid arthritis (RA). Studies have shown that gut microbiota alters during the pre-RA window, which leads to mucosal barrier dysfunction and skewed Th17/Treg balance [88]. IL-17, secreted by Th17 cells, promotes the development of RA, while IL-10 and TGF-β1, which are secreted by Treg cells, control the progression of RA. SCFAs, mainly butyrate and propionate, restore the intestinal barrier and rebalance Treg/Th17 cell ratios. Butyrate reduces arthritis severity by promoting anti-inflammatory IL-10 production while inhibiting pro-inflammatory IL-17. SCFAs activated GPCRs to induce IL-10 secretion and inhibit histone deacetylases to inactivate NF-κB and reduce inflammatory cytokines [89]. A study on humanized mice with collagen-induced arthritis found that *Prevotella histicola*, isolated from the human duodenum, could be a novel bacterial therapeutic for rheumatoid arthritis. *P. histicola* restored dysbiotic gut microbiota within 2 weeks in the humanized mice with the production of acetate. This led to the expansion of the *Allobaculum* genus, a butyrate-producing bacterium, across gut sections. The increment in SCFA levels activates the expression of Treg cells, maintains gut immune homeostasis, and reduces pro-inflammatory cytokines. *P. histicola* also produces biotin and folate vitamins, offering a potential alternative to chemical folate supplementation in methotrexate-treated RA patients [90]. Another study on subcutaneous injection of gut-derived bacterial extracts found that a single injection of sonicated luminal contents from the cecum improved glucose control in lean mice for up to five weeks, while obese mice needed a second dose to achieve improved glucose control. This works only if the innate immune system can detect bacterial components through the NOD2-RIPK2 signalling pathway, which engages adaptive immunity through localized IgG responses in the lower small intestine [91]. Some of the recent studies on the effects of postbiotics on SARS-CoV-2 have indicated that postbiotics could be used as adjuncts in treating viral infections. They induce signal cascades that stimulate the phagocytosis of viral antigens by host macrophages and promote the secretion of mucin and antimicrobial peptides (AMPs), strengthening the gut barrier against viral infection. Human defensin-5, an AMP, has a high affinity for the ACE2 (angiotensin-converting enzyme 2) receptor, which is reported to compete with SARS-CoV-2 for receptor binding and thereby limit viral entry into cells [92]. The metabolic products of *L. plantarum* have been reported to demonstrate antiviral activity in SARS-CoV-2 by interfering with the function of protein S (spike protein), which is involved in viral entry into host cells [93]. Extracellular vesicles (EVs) derived from the probiotic strain of *E. coli* EcO83 have exhibited the potential for use in vaccination strategies, particularly in mucosal vaccination. Intranasal administration of EcO83-EVs has been shown to trigger inflammation-related genes, including NF-κB2, BCL3, SOD2, IL-6, and IL-10 following the EV’s interaction with nasal and lung tissues, alongside the activation of key innate immune receptors such as TLR2, TLR4, TLR5, NOD1, and NOD2. Airway macrophages engulfed these EVs, which activated the TLR4-MAPK-NF-κB signalling pathway and eventually resulted in the production of both pro-inflammatory and anti-inflammatory cytokines. The production of IL-10 controlled inflammation while supporting neutrophil recruitment within the lungs [94]. Intranasal administration of EcO83-EVs mitigated a key feature of asthma—airway hyperresponsiveness—as well as decreased airway eosinophilia, Th2 cytokine production, and mucus secretion [95]. These findings highlight the potential of bacterial EVs as versatile vaccine platforms, functioning both as antigen delivery vehicles and immune-activating adjuvants. An overview of immunomodulatory and therapeutic roles of postbiotics across host systems is represented in Table 1.

## 6. Postbiotics in Cancer Therapy: Emerging Microbial-Derived Antitumor Strategies

Postbiotics are gaining attention and being investigated for their potential to complement conventional chemotherapy by mitigating systemic toxicity or functioning as alternative anticancer agents. They control carcinogenesis by reducing inflammation, inducing selective cytotoxicity against tumour cells, and restricting tumour cell proliferation, thereby serving as adjuncts in chemotherapy and immunotherapy and enhancing antitumor effects. Activation of GPCR and suppression of histone deacetylases are the two pathways through which SCFA exerts its activity [96]. They induce apoptosis in tumour cells by disrupting membrane potential and enhancing the expression of GPCR molecules, mechanisms that have been predominantly characterized in controlled in vitro systems. Recent evidence established that the gut microbiome of colorectal cancer (CRC) patients is characterized by a marked enrichment of taxa within the phylum Bacteroidetes, particularly *Bacteroides ovatus* and *Parabacteroides distasonis*. In contrary, individuals without CRC typically exhibit higher abundances of Firmicutes, including beneficial commensals such as *Bifidobacterium adolescentis* and *Bifidobacterium longum*. This taxonomic shift underscores a CRC-associated dysbiosis profile in which Bacteroidetes expansion and the depletion of Firmicutes may contribute to tumour-promoting metabolic and inflammatory microenvironments [97]. Furthermore, postbiotics *from B. adolescentis* and *B. longum* have been shown to increase the expression of CYP1A1, a cytochrome P450 enzyme. Alteration of CYP1A1 expression via histone deacetylase prevents cancer cell proliferation by detoxifying the tumorigenic factors of the tumour microenvironment [98]. Postbiotics from *L. acidophilus* was shown to suppress the Wnt signalling pathway by upregulating SFRP-1 and SFRP-2 proteins, thereby preventing frizzled receptor activation. This inhibition limits downstream localisation of β-catenin and target gene expression, including MMP7 (Matrix Metalloproteinase-7), whose overexpression promotes ECM degradation, cancer cell migration, and metastasis [99]. Postbiotics derived from *L. plantarum* YYC-3 exert anti-proliferative effects on CRC cells by modulating the VEGF/VEGFR (vascular endothelial growth factor receptor) signalling pathway, as demonstrated in colorectal cancer cell-based experimental systems, which normally activates MAPK and AKT to induce MMP2 and MMP9 expression. Downregulation of VEGF leads to a decrease in the expression of MMP2 and MMP9, resulting in a reduction in cell proliferation and angiogenesis [100].

**Figure 2:**
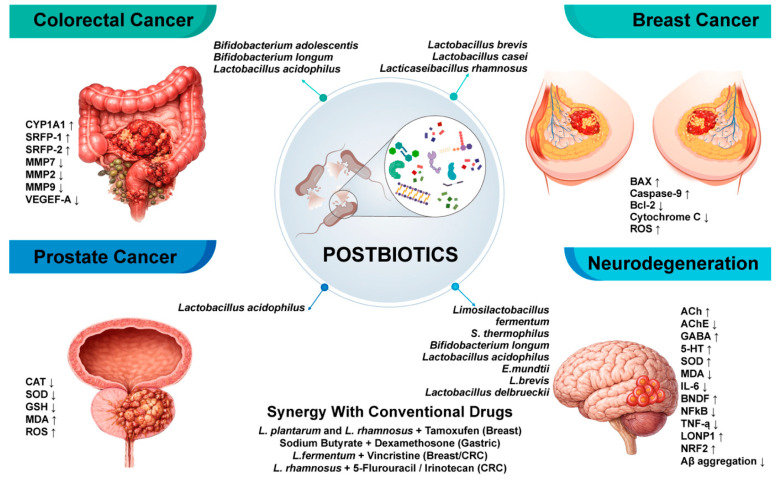
**Postbiotic-modulated mechanisms in downregulating cancer and neurodegeneration:** Probiotic bacteria such as *Bifidobacterium* and *Limosilactobacillus* spp. generate bioactive metabolites that influence oxidative stress, apoptosis, and inflammatory signalling in colorectal cancer (CRC), breast cancer, prostate cancer and neurodegenerative diseases. Postbiotics from beneficial strains may also act synergistically with conventional drugs, including tamoxifen, trastuzumab, irinotecan and vincristine, highlighting their potential as adjunct therapeutic agents.

In breast cancer cell lines, postbiotics from *Levilactobacillus brevis* and *L. casei* induced apoptosis by upregulating the expression of proapoptotic markers, BAX (Bcl-2 associated X protein) and Caspase-9. These postbiotics regulate the genes associated with the intrinsic and extrinsic apoptotic pathways, leading to an increase in mitochondrial membrane permeability, governed by the BAX and BAK (Bcl-2 homologous antagonist/killer) proteins [101]. Postbiotics derived from *Lactobacillus acidophilus* LA-5 reduce the activity of antioxidant enzymes, including catalase (CAT), superoxide dismutase (SOD), and glutathione (GSH), while increasing malondialdehyde (MDA) and reactive oxygen species (ROS) levels in prostate cancer cell lines [102]. This enhanced oxidative stress disrupts mitochondrial function and activates intrinsic, mitochondria-mediated apoptosis. In a study of 39 LAB strains, the post-fermentation media (PFM) and cell extracts (CEs) of some strains exhibited greater cytotoxicity toward cancer cells (Caco-2 and HeLa) than toward normal intestinal cells, highlighting selective anticancer activity under controlled experimental conditions. The Caco-2 cells were inhibited by PFM and CEs by generating oxidative stress through the production of hydrogen peroxide and the induction of ROS. The PFM of *L. plantarum* and *L. brevis* induced apoptosis by increasing the activity of caspases 3/7 and caspase 9, which led to the mitochondrial signalling pathway for apoptosis [103]. Pasteurized *Akkermansia muciniphila* and its outer membrane protein Amuc_1100 have been studied as potent immunomodulatory agents to prevent colitis-associated colorectal cancer (CAC). In murine CAC models, treatment resulted in delayed tumour formation, as well as reduced the expression of γH2AX and Ki67, which triggered the apoptosis of tumour cells and inhibited cell proliferation. Amuc_1100 promoted the activation of CD8^+^ T cells by increasing TNF-α induction and downregulation of the exhaustion marker PD-1 [104]. *L. reuteri*, another predominant probiotic bacterium, produces Indole-3-aldehyde (I3A) by metabolizing dietary tryptophan. I3A has been proven to enhance antitumor immunity by stimulating the production of IFN-γ in CD8^+^ T cells. Evidence from tumour-associated experimental systems indicates that *L. reuteri* translocates from the gut to tumours, colonizes the site, and produces I3A, which enhances IFN-γ production and improves cytotoxic T cell function [105]. Lysate extracts of *L. paracasei* have exhibited the potency to inhibit gastric cancer cells. The extracellular vesicles (EVs) of *L. paracasei* exert antiproliferative activity by decreasing HIF-1α expression as well as by affecting tumour metabolism [106]. Synergistic modulation of postbiotic molecules from *L. plantarum* and *L. rhamnosus*, in conjunction with antineoplastic drugs such as tamoxifen, displayed significant effects against breast cancer cells by increasing the rate of apoptosis [107]. Similarly, sodium butyrate, a key short-chain fatty acid postbiotic, demonstrates synergism with the corticosteroid, dexamethasone, reducing gastric cancer cell proliferation through apoptosis [108]. In synergy with the conventional cancer drug vincristine, *L. fermentum* postbiotics enhanced the cytotoxicity of vincristine on both breast cancer and colorectal cancer cell lines. The combinatorial treatment showed significant downregulation of anti-apoptotic genes (AKT, Bcl-2, and mTOR), while simultaneously upregulating pro-apoptotic genes (PTEN, BAX, Caspase-3/8/9, Fas, and IkB) [109]. Lyophilized postbiotics from *L. rhamnosus* GG also demonstrated synergy with 5-fluorouracil and irinotecan to induce cell cycle arrest in G2/M phase, exerting a cytostatic effect [110]; these findings establish postbiotics as multifaceted modulators of carcinogenesis, offering substantial potential to complement or enhance conventional anticancer therapies, although the majority of current evidence is derived from in vitro and preclinical studies, with limited validation in human clinical settings (Figure 2).

## 7. Restoring Neurotransmitter Homeostasis and Reducing Oxidative Stress in Cognitive Decline: The Neuroprotective Mechanisms of Postbiotics

Neurodegenerative diseases represent a heterogeneous group of disorders characterized by the progressive loss of structure or function of neurons, leading to severe cognitive, motor, and psychiatric impairments [111]. These conditions, including Alzheimer’s disease, Parkinson’s disease, and amyotrophic lateral sclerosis, are often associated with protein misfolding, neuronal cell death, and neuroinflammation, for which effective control therapies remain ineffective [112]. Gut dysbiosis significantly contribute to neuroinflammation and microglial activation, thereby stimulating neurodegenerative processes through various mechanisms including altered chemokine and cytokine signalling, leading to chronic neuroinflammation, and the progression of neurodegenerative disorders is a relationship supported by both experimental models and emerging clinical observations [113]. Recent studies have discussed the critical role of postbiotics in mediating beneficial host responses beyond the direct actions of live microorganisms [114]. Specifically, postbiotics can regulate inflammation and immunological responses, influence the aggregation of α-synuclein, and impact the neurotoxicity of proteins, even if their primary targets are distinct proteins [115]. The existence of SCFAs at a higher concentration in the cerebrospinal fluid of humans than in the peripheral blood suggests they may be useful as therapeutic agents [116]. This direct access to the brain highlights the profound implications of gut microbiota-derived metabolites on neurodegenerative processes; for instance, butyrate has shown to influence alpha-synuclein aggregation, reduce amyloid-beta and tau pathologies, and modulate neuroinflammation, thereby offering a multifaceted approach to combating neurodegenerative conditions [117]. In addition to having an impact on the protein pathologies, these compounds are important for maintaining several key functions of the brain through regulating neuroinflammation, blood–brain barrier integrity, and plasticity [118]. The neuroprotective effects of butyrate are further mediated through its activation of specific G-protein-coupled receptors, such as FFAR3 and GPR109a, and its potent inhibition of histone deacetylases [119]. Similarly, propionic acid exerts its effects by binding to GPR41 located on brain endothelial cells, conferring antioxidant protection of the blood–brain barrier via the NRF2 pathway [120]. Acetate is also important for the anti-neuroinflammatory actions of SCFAs through activation of GPR41 and GPR43, as it inhibits the production of several pro-inflammatory cytokines including TNF-α and IL-6. Furthermore, acetate plays a decisive role in supporting the integrity of the blood–brain barrier through the stimulation of tight junction protein synthesis [121]. Growing evidence establishes that specific postbiotic formulations exert neuromodulatory effects by exhibiting cholinergic, monoaminergic, and excitatory–inhibitory signalling pathways, while concurrently enhancing antioxidant defences and attenuating neuroinflammatory responses. Significant improvement in mice’s learning and memory was demonstrated with the consumption of powdered *L. fermentum* IOB802 postbiotics, highlighting functional outcomes in transgenic Alzheimer’s disease models. These effects were accompanied by increases in acetylcholine levels in the cortico-hippocampal region and downregulation in the activity of the acetylcholinesterase enzyme, both of which reflect normalization of cholinergic neurotransmitter function. Moreover, these processes were accompanied by the normalization of GABAergic and serotonergic neurotransmitter function and the reestablishment of balance between the excitatory–inhibitory systems and the functions of monoaminergic neurotransmitters (5-HT, DA, NE) in terms of cognitive damage. Moreover, the observed increase in superoxide dismutase and reduction in malondialdehyde levels reflect a substantive attenuation of oxidative stress, thereby preserving neuronal integrity and mitigating the progression of cognitive APP/PS1 mice to increase colonic propionic acid levels. Treatment with this postbiotic also elevated hippocampal IL-6 expression, enhanced microglial-mediated Aβ clearance, and upregulated both brain-derived neurotrophic factor (BDNF) and superoxide dismutase (SOD), augmenting synaptic resilience and antioxidant capacity [122]. Additionally, postbiotic lysates from *Bifidobacterium longum* and *L. acidophilus* attenuate Cu^2+^/Zn^2+^-induced Aβ-40/42 aggregation and reduce APPTG-derived terminal Aβ species, thereby modulating metal-driven oxidative stress and mitochondrial dysfunction as demonstrated in controlled biochemical and cellular aggregation models. Exercise regimes along with postbiotic interventions further suppress NF-κB-mediated neuroinflammation, lower ROS production, and enhance mitochondrial proteostasis via upregulation of LONP1 [123]. Similarly, postbiotics from *L. delbrueckii* subsp. *lactis* CRL581 and *Levilactobacillus brevis* CRL2013 significantly reduce IL-6 and TNF-α expression, counteracting microglial Aβ_1_–_42_-induced inflammatory signalling implicated in Alzheimer’s disease progression, with evidence derived from microglial activation and neuroinflammation models [124] (Figure 2).

Despite promising preclinical findings, the clinical translation of postbiotics or SCFA-based interventions for neurodegenerative diseases faces significant challenges due to the current lack of robust, long-term human studies and the slow, indirect nature of gut microbiome modulation. There is also wide variability among commercially available products both in their composition and stability and therefore will pose challenges for the application of these interventions with respect to the accuracy of controls. Furthermore, there are many variances in methodologies used in various bioinformatics studies, which make it difficult to effectively compare results, making further progression of this field of research very challenging due to the quality of evidence presented [125]. To overcome these limitations, rigorous and standardized methodologies must be developed, including large-scale human RCTs to validate the use of microbiota as biomarkers and to elucidate the precise means by which microbiota affect drug pharmacokinetics/pharmacodynamics in patients with neurodegenerative diseases [126]. Future research should leverage multi-omics approaches, including metabolomics, proteomics, and transcriptomics, to unravel the intricate mechanisms underlying postbiotic based therapies and to distinguish responders from non-responders.

## 8. Postbiotic Interventions for Targeted Wound Healing, Photo-Protection, and Skin Barrier Restoration

In contrast to probiotics, postbiotics display reduced safety issues related to live cultures while offering practical advantages like improved wound healing and UV protection. Innovative therapeutic approaches for treating a range of dermatological conditions are represented by these microbiome-targeted treatments. In wound therapeutics, postbiotics function as both potent antimicrobial/antivirulence agents and active stimulants for host tissue repair. The topical application of heat-killed *Lactococcus chungangensis* CAU 1447 markedly accelerated the closure of excisional wounds in a clinically relevant diabetic murine model [127]. Additionally, in *S. aureus*-infected human keratinocytes, synergistic interventions using CFS from *Lactobacillus johnsonii* in conjunction with vitamin D have shown the ability to improve cellular migration and suppress the pro-inflammatory cytokine interleukin-6 (IL-6), highlighting enhanced wound repair dynamics in controlled in vitro systems [128]. The gut–skin axis is a bidirectional communication pathway that suggests cutaneous homeostasis and gastrointestinal health are potentially linked, with gut dysbiosis often connected to dermatological pathologies. Butyrate, produced by some commensal bacteria such as *Faecalibacterium prausnitzii*, *Akkermansia muciniphila* and *Prevotella*, is one of the chief mediators of the crosstalk. However, there are far-reaching benefits of butyrate normalization, as it possesses the ability to modulate immune disorders, epigenetic pathways, and cytokine balance. Further, it also has a role as a histone deacetylase (HDAC) inhibitor and serves as a protective mechanism for atopic dermatitis (AD) as well [129]. Moreover, *L. gasseri* BNR17′s CFS has been proven as a powerful bioactive agent with both hypopigmenting and antioxidant qualities. By lowering tyrosinase activity, CFS efficiently prevents both extracellular and intracellular melanin synthesis without causing cytotoxicity, highlighting enhanced wound repair dynamics in controlled in vitro systems. This anti-melanogenic effect is mechanistically explained by metabolites like proline-serine, which inhibit pigmentation by activating the ERK phosphorylation pathway and downregulating tyrosinase and microphthalmia-associated transcription factor (MITF) [130]. Moreover, in the context of a clinical trial conducted over three weeks on the topical use of the *Epidermibacterium keratini* EPI-7 ferment filtrate of the postbiotic product EPI-7, a considerable structural improvement in facial skin was observed [131]. Additionally, in diabetic rats, the ethyl acetate extract (EAE) from *Lactobacillus plantarum* 2034, when topically applied, showed a clear ability to disrupt biofilms of methicillin-resistant *Staphylococcus aureus* (MRSA) by entirely clearing MRSA load on day 12 and significantly validating anti-infectivity. At a mechanistic standpoint, EAE doubled concentrations of anti-inflammatory cytokine IL-10 around wounds and normalized IL-6 [132].

**Figure 3:**
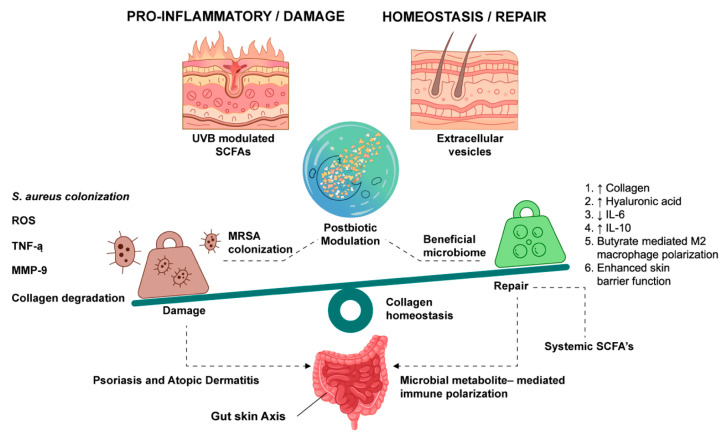
**Influence of postbiotic metabolites in maintaining skin homeostasis:** Skin dysbiosis represented by *Staphylococcus aureus* colonization, reactive oxygen species (ROS) production, and collagen degradation contribute to inflammatory skin disorders such as psoriasis and atopic dermatitis. Conversely, postbiotic-derived metabolites, including short-chain fatty acids (SCFAs) and extracellular vesicles, restore microbial balance and immune regulation by promoting M2 macrophage polarization, collagen synthesis, and barrier repair by upregulating hyaluronic acid, and IL-10, thereby maintaining gut–skin axis homeostasis.

Similarly, extracellular vesicles derived from *L. paracasei* (LpEVs) are efficiently internalized by human keratinocytes and fibroblasts, where they attenuate TNF-α-driven skin inflammation by suppressing key mediators such as MMP-1, IL-6, and IL-8. In three-dimensional full-thickness skin models, LpEVs further reverse epidermal thinning and restore dermal collagen production, highlighting their potential as cell-free anti-inflammatory and anti-ageing interventions [133]. The human skin bacterium, *Micrococcus luteus*, has emerged as a novel source of potent postbiotics, in addition to the conventional *Lactic Acid Bacteria* (LAB). By increasing genes associated with hyaluronic acid production, skin barrier strength, and cell growth, culture filtrates from the *M. luteus* YM-4 strain demonstrate broad advantages for skin health. Furthermore, by enhancing fibroblast mobility and reducing collagen degradation, this postbiotic filtrate protects against UV-B rays and accelerates wound healing. *M. luteus*-derived postbiotics can “reset” the gene activity of stressed or dehydrated cells to a normal state, as demonstrated by transcriptomic (RNA-sequencing) analysis, confirming their potential in advanced cosmetics and skin care [134]. Recent developments have enriched the postbiotic arsenal with *P. acidophilus*, leveraging the principles of photoprotection and regenerative repair. *P. acidophilus* LS cell-free supernatant (CFS-LS) possesses a broad-spectrum dermatological mechanism, uniquely interfering with melanogenesis, mediated through the PKA/CREB and MAPK pathway. Additionally, to its depigmentation properties, CFS-LS is an exceptionally potent photoprotector, stimulating the production of collagen Type I in conjunction with the replenishment of Nrf-2/HO-1 antioxidant pathways [135]. Similarly, postbiotics derived from *A. muciniphila* YGMCC2602 have also proved to be highly efficient at elevating the integrity of skin, including lysates and EVs. These products successfully decreased UVB-induced apoptosis/oxidative stress while increasing the expression of key basement membranes and hydration genes like COL17A1 and HAS1. These findings are also affirmed by clinical assessment studies, where *A. muciniphila* lysates were found to decrease erythema and TEWL, making this bacterium a prime candidate for inducing anti-ageing properties as well as increasing skin barrier integrity [136]. Similarly, postbiotic preparations from *Lacticaseibacillus paracasei* BGP1 exert potent bactericidal activity against *Pseudomonas aeruginosa* and *Acinetobacter baumannii* through irreversible membrane damage, positioning these metabolites as promising alternatives to conventional antibiotics for the management of wound infections [137]. Recently, there were also growing perspectives considering the role of postbiotics to help prevent UV-induced skin degeneration and therefore to expand the potential of therapeutic applications of postbiotics beyond wound healing. In biochemical and cell-based experimental systems, postbiotics extracted from fermented sources of *Cucurbita pepo* using *Limosilactobacillus* and kombucha cultures enhanced the antioxidant and anti-inflammatory attributes of naturally derived bioactive substances, producing extracts capable of scavenging reactive oxygen species, down-modulating pro-inflammatory cytokines such as TNF-α and IL-6, as well as inhibiting the activity of tyrosinase, thus exhibiting both anti-aging and skin-brightening properties with a high degree of cosmetic stability [138]. Strains including *L. plantarum* MG989 and *L. fermentum* MG5368 demonstrate efficacy in ameliorating UVB-induced skin barrier dysfunction, reducing transepithelial water loss and epidermal thickening [139], while *Latilactobacillus sakei* Wikim0066 regulates matrix metalloproteinase expression to preserve extracellular matrix integrity [140]. A brief illustration showing the effects of postbiotics on healthy vs. damaged skin is represented in Figure 3.

## 9. Postbiotics in Aquaculture and Sustainable Animal Production Systems

Aquaculture and livestock production are integral components of global food security, particularly in meeting the increasing demand for animal-derived protein. However, such production methods have become a significant challenge as they lead to serious environmental concerns, including water pollution, greenhouse gases, and widespread use of antibiotics, which are a common cause of antimicrobial resistance. The use of postbiotics has been explored as an alternative to antibiotics, especially with regard to their effects on the immune system, antioxidants, and resistant pathogens [141]. The anti-bacterial compounds, also known as strains, are reported to have inhibitory effects on numerous pathogens, as well as enhance the intestinal barrier and improve the efficiency of feeding, as characterized through controlled pathogen inhibition and biofilm-based assays [142].

Postbiotics from *B. subtilis* and *B. amyloliquefaciens* were compared, and *B. subtilis* postbiotics displayed stronger antibacterial, antivirulence, anti-biofilm, and antioxidant activities. These properties resulted from strain-specific metabolites interfering with pathogen survival and virulence. *B. subtilis* postbiotics also stimulated immune responses in *Labeo rohita* head-kidney leucocytes by promoting myeloperoxidase activity, leukocyte proliferation, nitric oxide and superoxide production, and immune gene expression (IL-1β, IL-10, IFN-γ, TNF-α), integrating both cellular immune assays and organism-level responses [143]. The postbiotics derived from *Weissella cibaria* isolates of rainbow trout (*Oncorhynchus mykiss*) and Nile tilapia (*Oreochromis niloticus*) exhibited strong antagonism against *Aeromonas salmonicida* subsp. *Salmonicida*, whereas *Yersinia ruckeri* growth was only partially inhibited, exhibiting the lowest inhibition values. Thirteen isolates of *Weissella cibaria* were tested; among them, strains PTB8 and NTM10 showed maximum activity, indicating that potency is strain-specific and supporting their potential as alternatives to antibiotics in aquaculture [144].

Postbiotics upregulate the expression of immune-related genes that florfenicol suppresses, thereby stimulating host defence mechanisms rather than exerting direct antibacterial effects. In the gastrointestinal tract, 1–2% postbiotics showed induction of elongation in intestinal microvilli and epithelial cell integrity, which is related to improved nutrient absorption and contributes to an enhanced physical barrier against invading pathogens. Collectively, through microbial metabolites that act as stimuli for the antioxidant and immune systems and enhance intestinal morphology, postbiotics function as functional fish feeds in reducing antibacterial use during *Macrobrachium nipponense* culture, demonstrating efficacy under in vivo aquaculture feeding conditions [145]. Additionally, extracellular products (ECPs) from *Shewanella putrefaciens* grown on aquafeed media enriched with microalgae/cyanobacteria as a microbial substrate were non-cytotoxic to fish cell lines and demonstrated enzymatic and antioxidant activities, highlighting the potential of these microbial substrates for the generation of novel postbiotics in fish-derived in vitro systems prior to in vivo application [146]. Similarly, dietary supplementation of *Vibrio proteolyticus* extracellular products led to an increase in the diversity of gut microbiota as well as the number of goblet cells, indicating an enhanced barrier integrity and function. Notably, these adaptations were not linked to inflammation or any signs of tissue damage, indicating that postbiotic-induced changes are beneficial rather than stress-related [147]. Similarly, postbiotics produced by *Weissella cibaria* strains when included in the diet of rainbow trout (*Oncorhynchus mykiss)* resulted in an increased level of lactic acid bacteria in the rainbow trout intestinal tract and upregulated pro-inflammatory cytokines IL-1β and downregulated IL-10, IL-8, INF-γ, and TNF-α, reflecting systemic immune modulation in vivo [148].

There is growing evidence that postbiotics are a significant contributor to improved efficiency of feed conversion in agriculture. For example, yeast postbiotics, like SCFP produced by DVAQUA, create significant improvements in feed conversion and protein efficiency ratios of shrimp; this likely occurs through enhancement of digestive enzyme activity and nutrient absorption. The functional potential of postbiotics is not limited to aquaculture, as *B. subtilis*-derived postbiotics can improve immunity by increasing serum levels of total proteins, albumin, antibody production, calcium, and phosphorus levels in tibia of poultry [149,150]. In addition, the use of *B. subtilis* postbiotics increased body weight gain, feed efficiency, and meat yield without significantly affecting intestine morphology or carcass composition. Environmental benefits have also been observed, as postbiotic-fed birds showed reduced ammonia emissions and lower *Salmonella* prevalence in excreta, potentially due to the proliferation of beneficial *Lactobacillus* spp. that competitively inhibit pathogens [151]. Exopolysaccharides (EPS), produced by lactic acid bacteria, was reported to enhance the survival of beneficial bacteria in livestock by increasing tolerance to gastrointestinal stress and supporting mucosal biofilms. Additionally, EPS can be used to inhibit pathogenic organisms such as *E. coli*, *Salmonella*, and *Clostridium perfringens* through growth inhibition, blocking adhesion, biofilm degradation, and coaggregation [152]. In swine models, the EPS produced by LAB directly modulates the activation pathways of TLR2/TLR4 and NF-κB with substrate-dependent variability in growth EPS production (dosage-dependent EPS effects), proving their potential immunomodulatory activity in farm-raised animals [153].

Postbiotics from *L. plantarum* (RI11, RS5, UL4) mitigated heat stress in broilers by enhancing blood levels of total antioxidant capacity, catalase, and glutathione, all of which are imperative enzymes that neutralize harmful free radicals. They also reduced inflammatory stress proteins such as alpha-1-acid glycoprotein and ceruloplasmin, indicating reduced systemic stress compared with both unsupplemented and antibiotic-treated birds. From the technological meat quality perspective, postbiotics increased breast meat pH into the desirable range, which is associated with improved water-holding capacity and juiciness in broiler meat [154]. In broilers, dietary *Lactiplantibacillus plantarum* postbiotics increased antioxidant enzymes (SOD, glutathione peroxidase), mucin production, and expression of MUC2 and occludin, while promoting colonization by beneficial gut bacteria; mechanistically, this combination of increased mucus production and enhanced tight junction integrity and improved redox balance underpinned improved growth performance and feed conversion comparable to that achieved with antibiotic growth promoters [155].

**Figure 4:**
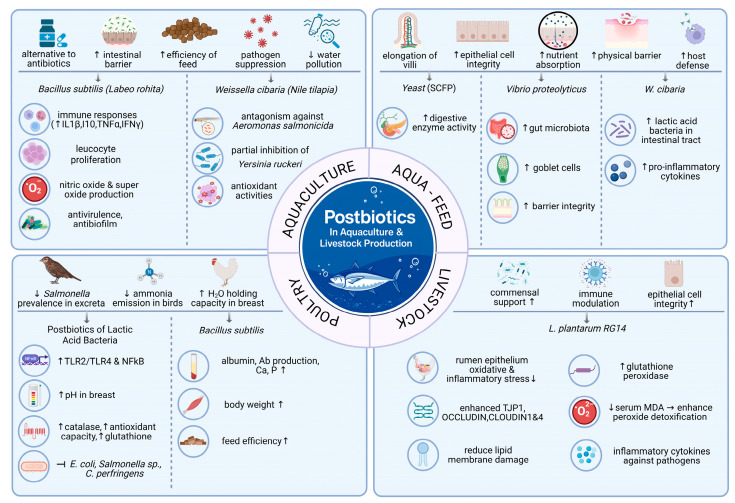
**Influence of postbiotics in aquaculture and livestock production:** Postbiotics from *Bacillus subtilis*, *Weissella cibaria*, and yeast (SCFP) enhance immune responses (IL-1β, TNF-α), digestive enzyme activity, gut microbiota balance, epithelial integrity, and suppress pathogens such as *Aeromonas salmonicida*. In livestock and poultry, lactic acid bacteria and *B. subtilis*-derived postbiotics alleviate growth performance, antioxidant ability, tight junction integrity, and reduce pathogen load and ammonia emissions, contributing to better sustainability.

The effect of *Lactobacillus plantarum* postbiotic was also evaluated in post-weaning lambs. Dietary postbiotic from *Lactobacillus plantarum* RG14 was selected because it had the strongest ABTS antioxidant activity in vitro, indicating superior free radical-scavenging capacity. In post-weaning lambs, 0.9% RG14 increased glutathione peroxidase (GPX) in serum and rumen and lowered serum MDA, thereby enhancing peroxide detoxification and reducing lipid membrane damage. It enhanced the genes of tight junctions in the rumen epithelium, such as TJP1, occludin, claudin-1, and claudin-4, thereby tightening the barrier and limiting the passage of toxins and microbes into the blood. Collectively, these effects protect post-weaning lambs from oxidative and inflammatory stress, improving livestock production [156]. Apart from local intestinal effects, postbiotics can act systemically by regulating metabolism/hormonal pathways in broilers. Encapsulated postbiotics, used alone or in combination with insulin, augmented meat yield, serum antioxidant levels, growth hormone and Insulin-Like Growth Factor (IGF) mRNA expression, reflecting coordinated control of immune, redox, and growth processes, respectively [157]. Together, these mechanisms, which include microbiota modulation reinforcement, immune modulation, antioxidant protection, and systemic endocrine effects, account for why postbiotics are now increasingly recognized as multifunctional instruments for improving performance, health, and product quality in modern livestock production systems. In addition, extracellular preparations from marine organisms and *Vibrio* spp., together with LAB exopolysaccharides, modify intestinal microbial flora by boosting beneficial microbes with normal cytokine/TLR/NF-κB pathways without inducing inflammatory injury, which implies a safe strategy in engineering microbial flora with non-viable preparations. However, the applicability of such results into regular aquaculture and livestock production remains limited due to the lack of standardized, large-scale field trials in various farming methods. A consolidated representation of the applications of postbiotics in aquaculture is represented in Figure 4.

## 10. Postbiotics for Biostimulation and Biocontrol in Sustainable Agriculture

Plant biostimulants and biocontrol agents have been identified as a plausible tool in realizing a sustainable agri-food system that serves as a sustainable alternative to agrochemicals. The role of these agents in improving plant growth, stress tolerance, and protection of crops against a variety of stress agents can hardly be overemphasized. As the agricultural industry is being increasingly pressed to reduce chemicals while protecting the environment, there has been intensified research into bio-based approaches. Bioactive compounds and metabolites of microbial origin in the form of cell-free exudates can be used as postbiotics for increased plant growth and development and eco-friendly processes. Plant-growth-promoting rhizobacteria (PGPRs), plant-growth-promoting fungi (PGPFs), endophytes, mycorrhiza, algae, and biocontrol agents boost plant growth by increasing seedling biomass, root architecture, shoot biomass, yield, and nutrient uptake [158]. Cell-free exudates from these plant-growth-promoting microbes (PGPMs) can act as postbiotics. For example, organic acid-rich exudates of PGPMs can solubilize inorganic phosphate and potassium ions, enhance root and shoot lengths, chlorophyll content, leaf area, flowering, and yield. Exudates containing phytases and acid phosphatases facilitate the mineralization of organic phosphorus, thereby improving nutrient-use efficiency. Exopolysaccharides enhance stress tolerance by aiding root water retention and modulating osmolytes and stress-responsive genes under heat and drought stress conditions [159].

As biocontrol agents, cell-free supernatant (CFS) metabolites such as iturins, fengycins, and surfactins from *Bacillus* spp. can result in up to a 71% reduction in *Rhizoctonia solani* infestation on potato and effectively control multiple foliar diseases in tomato, strawberry, and pepper [160]. Amphiphilic bacterial lipopeptides, such as those produced by plant-beneficial *Bacillus* and *Pseudomonas* spp., can insert into pathogen cell membranes and disrupt integrity, leading to cellular leakage and pathogen inhibition, and can also function as elicitors of plant defence signalling [161]. The bioactive molecules produced by beneficial microbes can induce systemic resistance in plants through mechanisms involving jasmonic acid and ethylene, which cause reinforcement of the cell wall and accumulation of defence-related proteins such as pathogenesis-related (PR) proteins at sites of infection [162]. Similarly, *Bacillus amyloliquefaciens* DHA6 produces cyclic lipopeptides, namely iturins, surfactins, bacillomycin, pumilacidin, and syringfactin, which have direct antifungal activity against *Fusarium oxysporum f*. sp. *niveum* [163]. It acts by extensively damaging mycelia and conidia and inhibits mycelial growth (up to 87%) and spore germination (94%), showing that CLPs have the potential to act as postbiotics. In watermelon, CLP pretreatment promotes growth and reduces *Fusarium* wilt severity by 55% and elicited immune responses by upregulating defence-related genes (PR1, PR2, PDF1.2), salicylic acid (ICS1, EDS5, PAL2), and jasmonic acid/ethylene (AOS, EIN2, CTR1) signalling pathways, thereby inducing systemic resistance. Rhamnolipids and fengycins from *Bacillus subtilis* disrupt pathogenic fungal hyphal structures, resulting in loss of mycelial structure and abnormal hyphal fusions in *Botrytis cinerea* and *Sclerotinia sclerotiorum* [164]. Jasmonic acid (JA) produced by *Pseudomonas* spp., *Bacillus* spp., and *Azospirillum* spp. triggers the systemic resistance against the caterpillar *Spodoptera exigua* via JA signalling-induced defence gene expression and anti-herbivore compounds [165].

Additionally, chitosan microparticles encapsulating indole-3-acetic acid (IAA) (EN-IAA) from *Stenotrophomonas maltophilia* PT53T cell-free supernatants significantly increased tomato seed germination by 80.8% (*p* < 0.05) at an optimal low concentration (15.88 μg/mL), while maximizing aerial part length (8.27 cm) and fresh weight (0.04 g) relative to control treatments. Low levels of IAA activate the TIR1/AFB auxin receptors, which activate the ARF transcription factors, promoting hypocotyl cell elongation and meristem proliferation via AUX/IAA degradation [166]. Bacterial production of auxin-like compounds (notably indole-3-acetic acid) activates auxin-responsive gene networks, including AUX/IAA and ARF transcription factors, resulting in enhanced lateral root initiation and root hair elongation [167]. Metabolites influencing ethylene (ET) metabolism act through ACC deaminase activity, lowering ACC levels, and thereby reducing ethylene signalling via receptors such as ETR1, which alleviates ethylene-mediated growth inhibition under stress [168]. In addition, some of these bacterial signalling molecules trigger induced systemic resistance via JA/ET signalling pathways, via regulators such as MYC2, in order to enable growth/defence gene expression regulation [169].

Microalgae and cyanobacteria produce a broad range of bioactive compounds, such as phytohormones (cytokinins and gibberellins), polysaccharides (including heteropolysaccharides), amino acids, and signal molecules, which, when used as biostimulators for plants, display pronounced plant nutrient uptake and plant hormone homeostasis-regulating effects [170]. For instance, cytokinins promote cell division via activation of type-B response regulator genes, and polysaccharide-based elicitors induce defence-linked enzymes like phenylalanine ammonia lyase (PAL), chitinase, and peroxidase, which increase defence metabolites under stress conditions [171]. Volatile organic compounds (VOCs) are low-molecular-weight, high-vapour-pressure secondary metabolites that can diffuse through soil and air without direct microbial contact with their targets, acting over relatively long distances in the rhizosphere [172]. *Streptomyces* spp. are prolific producers of VOCs, which have been shown to possess antimicrobial functions against phytopathogens such as *Rhizoctonia solani*, making them promising candidates for use as biofumigants to suppress soil-borne diseases and reduce reliance on synthetic pesticides [173]. In agriculture, microbial VOCs can be used as alternatives to chemical pesticides [174]; therefore, these compounds may be exploited as postbiotic metabolites for modulating plant physiological pathways and inhibiting pathogens without requiring active microbial colonization, suggesting a potential route for the development of VOC-based postbiotic formulations for crop protection. Plants synthesize ethylene from 1-aminocyclopropane-1-carboxylate (ACC), which can be cleaved by microbial ACC deaminase into α-ketoglutarate and ammonia, thereby limiting ethylene overproduction [175]. Inhibiting the overproduction of ethylene becomes necessary as excessive accumulation suppresses root development. ACC deaminase-producing microbes such as *Pseudomonas* spp., *Variovorax paradoxus*, *Rhizobium phaseoli*, and several actinomycetes reduce stress-induced ethylene and alleviate salinity stress. For instance, *Streptomyces* spp. PGPA39 enhanced tomato tolerance to salinity via ACC deaminase activity along with phosphate solubilization and IAA production, improving chlorophyll and water content [176].

**Figure 5:**
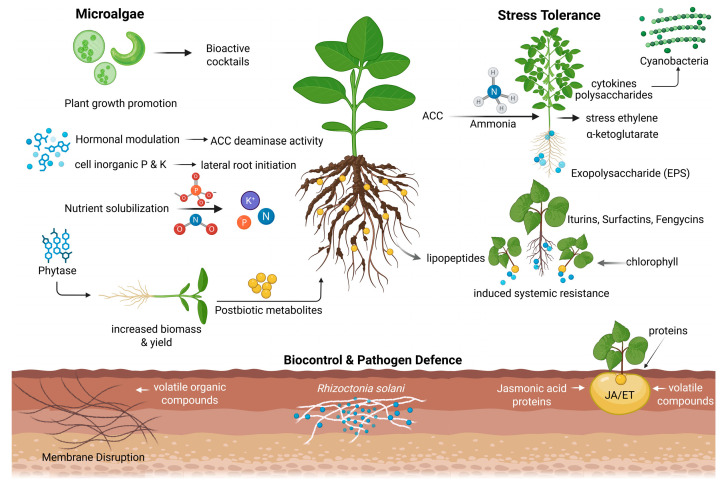
**Effects of postbiotics in plant biocontrol and pathogen defence:** Microalgae produce diverse bioactive compounds that enhance plant growth through hormonal modulation, nutrient solubilization, and enzyme-mediated processes such as phytase activity, cell wall degrading enzymes, thereby facilitating improved nutrient availability. These compounds stimulate ACC deaminase activity enhancing plant stress tolerance. Postbiotic metabolites also induce systemic resistance through jasmonic acid and ethylene (JA/ET pathways), along with the production of exopolysaccharides, lipopeptides, and other antimicrobial compounds. Additionally, volatile organic compounds and other antimicrobial metabolites induce pathogen suppression collectively, these promoting plant biomass, improved yield, enhanced stress tolerance, and biocontrol against phytopathogens.

There is increasing evidence of stable bacteriocins (such as enterocins produced by *Enterococcus faecium*) within a wide pH range and at high temperatures [177]. However, the environmental half-life of these compounds has not yet been established. Postbiotics was also found to have significant effects on the bacterioplankton communities of aquaculture impacting in both general and species-specific shifts in community structure and function [178]. However, the effect of postbiotics on beneficial and native microbial communities and persistence in the environment still needs to be determined. Surfactin, a cyclic lipopeptide biosurfactant from *Bacillus* spp., shows strong surface activity, antimicrobial effects, and relative safety. It can both stabilize food structures and actively protect against pathogens, while also exerting biological effects in the gut and systemic health. Surfactin also modulates gut microbiota, improving immune and barrier functions, linking its food use to broader human health benefits [179]. A schematic representation summarizing plant biocontrol mediating postbiotic mechanisms is shown in Figure 5.

## 11. Conclusions

In summary, this article aims to provide an accumulating body of evidence on the promising state of postbiotics as an alternative to live probiotics collectively in both physiological and ecological processes. It is crucial to understand the growing evidence of how prominent postbiotics such as short-chain fatty acids (SCFAs), extracellular vesicles (EVs), and antimicrobial peptides interact with important inflammatory and metabolic pathways like the NF-κB and MAPK cascades. In addition to their importance in systemic health, their significance as regulators of wound healing, functioning as dual-functional modulators that inhibit pathogenic biofilm and at the same time have regenerative functions that include collagen remodelling and activation of fibroblasts, remains significant. Postbiotics are an emerging area of research and are highlighted for their synergistic effect with existing drug therapies and selective cytotoxicity against cancer cell lines. In addition, postbiotics have been reported to be bioactive agents that can alter the dynamics of microbial communities in agricultural and aquacultural systems by suppressing microbial populations without altering the structure or function of the surrounding ecosystem. Thus, postbiotics are poised to play a central role in next-generation therapeutic and biotechnological innovations, bridging gaps between microbiome science, clinical translation, and sustainable applications.

Unfortunately, most of the current literature concerning postbiotics is based largely on correlative data and they are not adequately validated with respect to their effects in the host. A significant lack of pharmacokinetic and pharmacodynamic data for most of the postbiotic molecules adds complexity to this issue. Due to their heterogeneous nature, complexities arise in establishing a standardized approach to incorporate into therapeutic regimens. Although postbiotics have immense therapeutic possibilities, the implementation of these agents in clinical practice requires rigorous standardization. The inherent complexity of the postbiotic composition presents challenges in achieving batch-to-batch consistency; thus, sophisticated analyses involving metabolomics and proteomics, including liquid chromatography–mass spectrometry and high-performance liquid chromatography, as well as functional assays, are needed to guarantee consistency. In parallel, adherence to controlled manufacturing processes and established regulatory guidelines will be essential to ensure product quality, safety, and scalability for clinical applications. Moreover, the factor of dose dependence and variability in the host/ecological systems further complicates the establishment of an accepted standardized methodology for their formulation.

## 12. Future Perspectives

Future studies should involve integration of microbiota metabolite signatures to be used as predictive biomarkers for severe immune-related adverse events during treatment with immune checkpoint inhibitors. These integrative models, which include specific bacterial species, have indicated high accuracy for stratifying patients who may be at risk of adverse events prior to treatment initiation. Advanced analytical tools such as the Virtual Twins method, in combination with distance-based machine learning, have been found to facilitate the identification of the microbial subgroups that correlate with different clinical outcomes. Such computational models may potentially allow the identification of underlying patterns of heterogeneity in the microbiome that could impact drug response or toxicity in the future. The integration of machine learning with the microbiome is one of the significant innovations that have led to the development of predictive and personalized medicine in the fields of precision oncology and microbiome therapeutics. While this innovation has huge potential for application, the integration of artificial intelligence with the microbiome in routine oncology practice faces a number of barriers, such as the lack of standards in data acquisition and processing, differences in sequencing technologies, scarcity of large and annotated patient cohorts, and problems with model validation and interpretability.

However, recent evidence has also shown that the gut-derived microbial metabolite, indole-3-propionic acid can augment immune checkpoint therapy by maintaining progenitor-exhausted CD8^+^ T cells through epigenetic regulation by H3K27 acetylation. It can be anticipated that in the future, the role of the microbiome-derived metabolites may be utilized as therapeutic agents to maintain anti-tumour immune responses and overcome the problem of immune exhaustion. Emerging evidence from pharmacomicrobiomics highlights the critical role of the microbiome in modulating drug response and toxicity in hematological malignancies. Microbial enzymes such as β-glucuronidase and metabolites like butyrate influence drug metabolism and mucosal immunity during intensive treatments, including allogeneic hematopoietic cell transplantation. Future strategies may involve microbiome and metabolite profiling to identify patients at risk of complications such as graft-versus-host disease, enabling targeted modulation of microbial metabolism to improve therapeutic safety and efficacy. Moreover, microbiota-derived extracellular vesicles could also potentially function as a tool for simultaneously managing intestinal inflammation and its systemic effects. The design and development of new synthetic biology methods for utilizing microbes have also been used for enhancing cancer immunotherapy. An engineered strain of *Clostridium butyricum* that can provide more tryptophan while inhibiting the activity Indoleamine 2,3-dioxygenase (IDO) also enhances the CD8^+^ T cells’ antitumor immune response. These findings indicate the capacity to use programmable commensal organisms to develop better immune checkpoint therapies for cancer immunotherapy in the future Bacteria–nanomaterial hybrid platforms are also emerging as innovative systems for targeted cancer immunotherapy. By exploiting the natural tumour-homing capacity of *Salmonella typhimurium* and related engineered bacteria, these hybrids can transport therapeutic nanomaterials into hypoxic and poorly vascularised tumour regions. Future developments integrating synthetic biology and nanotechnology may enable highly controlled, site-specific immunomodulation with reduced systemic toxicity. Future research should also prioritize advanced delivery platforms, such as hydrogel-based systems and nanoparticle encapsulation, to enable targeted and sustained release in complex biological environments. In parallel, exploration of marine-derived postbiotics and integration of sustainable “blue biorefinery” models offer promising avenues to expand the postbiotic repertoire. To conclude, as regulatory frameworks continue to mature, postbiotics are well-positioned to emerge as a cornerstone of next-generation microbiome-based strategies that bridge precision medicine and sustainable biotechnology.

## Figures and Tables

**Table 1:** Immunomodulatory and therapeutic roles of postbiotics across host systems. The arrows indicate the direction of change: ↑ increase/enhancement; ↓ decrease/reduction.

Postbiotic Component	Primary Mechanism(s)	Immune/ Physiological Effects	Disease/Application	References
Microbe-associated molecular patterns (MAMPs)	PRR engagement; NF-κB and MAPK/AP-1 activation	Regulation of innate and adaptive immunity; restore Th1/Th2 balance; ↑ IgA production	Immune homeostasis; infection resistance	[16,79]
Short-chain fatty acids (SCFAs)	GPCR activation; HDAC inhibition; mitochondrial regulation; modulate NF-κB	↓ Pro-inflammatory cytokines; ↑ IL-10; enhanced Treg differentiation; improved barrier integrity	IBD, rheumatoid arthritis, metabolic disorders	[80,81,88]
Exopolysaccharides (EPS)	PRR-mediated signalling; macrophage activation	↓ Pro-inflammatory cytokines; immune cell differentiation; microbiome restoration	Inflammatory bowel disease	[80]
Cell-free supernatants (CFSs)	Antimicrobial and antioxidant activity; macrophage and NK cell activation	Pathogen inhibition: cytokine modulation, enhanced innate immunity	Infection control; stress-induced immune suppression	[65]
Lipoteichoic acid	PRR engagement (TLRs)	Fine-tuning of innate immune activation without excessive inflammation	Inflammatory and immune-mediated disorders	[81]
β-Glucans	PRR interaction; NF-κB/MAPK activation; complement activation	Enhanced phagocytosis; improved pathogen clearance	Vaccine adjuvants; infectious diseases	[82]
Flagellin	TLR5 activation; type I interferon signalling; IgA induction	Reduced microbial encroachment; attenuation of inflammation and obesity	Obesity, gut inflammation, hepatic fibrosis	[83]
Extracellular vesicles (EVs)	Activation of TLR2/4/5, NOD1/2;	↑ NF-κB2, BCL3, SOD2, IL-6, IL-10; reduced airway hyperresponsiveness, ↓ eosinophilia and Th2 cytokine	Mucosal vaccination; asthma	[94,95]
Postbiotic metabolites (SCFAs, indole metabolites, muramyl dipeptide)	Viral antigen phagocytosis; AMP induction; ACE2 competition	Reduced viral entry; enhanced antiviral defence	SARS-CoV-2 and viral infections	[92,93]
Fermented formula from LAB-derived postbiotics	Barrier enhancement; mucin induction; cytokine modulation	Improved intestinal homeostasis; reduced infection rates	Paediatric nutrition and immunity	[86,87]
Gut-derived bacterial extracts	NOD2–RIPK2 signalling; IgG-mediated adaptive immunity	Improved glucose control; metabolic homeostasis	Metabolic disorders; obesity	[66,91]

## Data Availability

No new data were created or analyzed in this study. Data sharing is not applicable to this article.

## Abbreviations

The following abbreviations are used in this manuscript:

ACP	Acid Phosphatase
AHL	N-acyl Homoserine Lactone
AI	Artificial Intelligence
AKP	Alkaline Phosphatase
AMP	Antimicrobial Peptide
AMPK	AMP-activated Protein Kinase
AP-1	Activator Protein-1
AhR	Aryl Hydrocarbon Receptor
BBB	Blood–Brain Barrier
BDNF	Brain-Derived Neurotrophic Factor
BLIS	Bacteriocin-Like Inhibitory Substance
CAT	Catalase
CE	Cell Extract
CFS	Cell-Free Supernatant
CRC	Colorectal Cancer
CREB	cAMP Response Element-Binding Protein
ECM	Extracellular Matrix
EPS	Exopolysaccharides
EVs	Extracellular Vesicles
GPCR	G Protein-Coupled Receptor
HDAC	Histone Deacetylase
IL	Interleukin
LAB	Lactic Acid Bacteria
LTA	Lipoteichoic Acid
MAMPs	Microbe-Associated Molecular Patterns
MAPK	Mitogen-Activated Protein Kinase
MMP	Matrix Metalloproteinase
MRSA	Methicillin-Resistant Staphylococcus aureus
NF-κB	Nuclear Factor Kappa B
OMVs	Outer Membrane Vesicles
PRRs	Pattern Recognition Receptors
QQ	Quorum Quenching
QS	Quorum Sensing
ROS	Reactive Oxygen Species
SCFAs	Short-Chain Fatty Acids
SOD	Superoxide Dismutase
STAT3	Signal Transducer and Activator of Transcription 3
TLR	Toll-Like Receptor
TNF-α	Tumor Necrosis Factor-alpha
Treg	Regulatory T Cells
UV	Ultraviolet
VEGF	Vascular Endothelial Growth Factor
